# Impact of Cone Beam Computed Tomography Dose in Pre-Surgical Implant Analysis

**DOI:** 10.2174/1874210601812010094

**Published:** 2018-01-31

**Authors:** Nabil EL Sahili, Ibrahim Nasseh, Antoine Berberi, Sandra David-Tchouda, Sophie Thoret, Thomas Fortin

**Affiliations:** 1 School of Dentistry, Lebanese University, Beirut, Lebanon; 2 Department of Oral & Maxillofacial Radiology, School of Dentistry, Lebanese University, Beirut, Lebanon; 3 Medico-Economic Evaluation Unit, University Hospital of Grenoble, Grenoble, France; 4 ThEMAS TIMC UMR CNRS 5525, Grenoble Joseph Fourier University, France; 5 Investigation Clinical Center of Grenoble, INSERM, Paris, France; 6 Department of Oral Surgery, Dental University of Lyon, University Claude Bernard, Lyon 1, France; 7 UJF-Grenoble 1 / CNRS / TIMC-IMAG UMR 5525, Grenoble, F-38041, France

**Keywords:** Cone-beam computed tomography, Implant-placement planning, Image quality, Radiation dose alteration, Posterior mandibular implant, Hemi Maxillary segments (HM)

## Abstract

**Objectives:**

Cone-Beam Computed Tomography (CBCT) produces vital information required for the accurate and prudent placement of dental implants. Lack of standardization between CBCT machines may result in unsafe patient exposure to harmful radiation; higher doses are not necessarily associated with improved image quality.

**Aim:**

The study aimed to assess the influence of low- and high-dose milliamperage settings on CBCT images for objective and subjective implant planning.

**Methods:**

Two dry skulls (4 hemi-maxillary segments of the maxilla and 4 hemi-maxillary segments of the mandible) were scanned under low (2 mA) and high (6.3 mA) dosage settings using a CBCT (Carestream CS 9300). Cross-sectional slices of both image qualities were evaluated by five expert clinicians, for image quality for implant planning and objective bone measurements.

**Results:**

There were no significant differences in bone measurements taken on high or low dose images (*p* > 0.05). In qualitative image assessments, assessment and image quality for almost all observers were independent of each other. For planning posterior mandibular implant placement, increased dosage improved concordance and kappa values between low and high dose images.

**Conclusion:**

Reduction in milliamperage did not affect diagnostic image quality for objective bone measurements and produced sufficient intra-rater reliability for qualitative assessment; therefore dose reduction can be achieved without compromising diagnostic decision- making.

## 1 INTRODUCTION

Cone Beam Computed Tomography (CBCT) is increasingly taking its place as a reliable radiological technique in the field of oral and maxillofacial radiology owing to the advantages it offers over traditional techniques such as panoramic radiography and medical Computed Tomography (CT). To the patient, the benefits include reduced radiation doses compared to conventional three-dimensional imaging techniques and the ability to adjust and limit the irradiated surface/field of view as diagnostically required, both resulting in lower patient exposure to harmful radiation [1]. To the practitioner, CBCT offers a cost-effective method for the visualization of neurovascular and osseous structures during pre-surgical treatment planning; allowing the assessment of bone volume, trabecular structure and the presence of pathology [1, 2].

When planning the placement of dental implants, CBCT offers information that is crucial to the selection of final implant size and location by allowing the clinician to assess the amount, density and quality of bone, ultimately allowing optimal implant placement in avoidance of vital structures such as the mandibular canal and inferior alveolar nerve, the mandibular posterior lingual undercut, and the maxillary sinus [3]. However, the frequency of radiographic examinations in dental practice and the lack of standardization in CBCT dosimetry have raised concerns over the indications, safety and clinical practice patterns in patient referral for CBCT both by the American Association of Oral and Maxillofacial surgeons and by the International Commission on Radiological Protection (ICRP) [4-6]. As with other radiological imaging modalities, the use of CBCT must satisfy the concept of “As Low As Reasonably Achievable (ALARA)” while maintaining sufficient imaging quality for appropriate diagnosis [5, 6]. However, there is a lack of published information on the appropriate methods to determine patient doses from CBCT equipment. The purpose of this study was to measure the impact of the reduction in the dose emitted by the CBCT on experienced clinicians’ ability to retrieve diagnostically useful information for pre-surgical treatment planning of dental implant placement

## 2 MATERIALS AND METHODS

### 2.1 Population

This study was conducted on two fresh cadaver skulls. Each skull was split into 4 Hemi Maxillary (HM) segments (1, 2, 3, and 4 according to the international nomenclature: 1) upper right maxillary; 2) upper left maxillary; 3) left mandibular and 4) right mandibular. The skulls were wrapped with clear plastic and fixed in position for subsequent CBCT imaging.

### 2.2 Image Acquisition

The CBCT machine used to acquire all images was the Carestream CS 9300 (Carestream Health Inc.). Given that effective radiation dose depends on the interplay of 4 parameters (kilo-voltage, milliamperage, exposure time and Dose-Area-Product (DAP)), the aim was to use two different milliamperage modes representing low dose and high dose radiation. Voltage was kept constant but exposure time was reduced by one third to half the time in conventional mode, at approximately 20 seconds.

Voltage was set at a fixed value of 78 kV, representative of the average range for most CBCT devices in France (60-90 kV), which provides low-contrast images but with greater shades of gray thereby allowing greater differentiation between slight variations in image quality. Two different settings were used to acquire low dose (2 mA) and high dose (6.3 mA) images. Using the standard DAP measure of dosimetry for dental radiology, exposure was 120 mGy.cm^2^ for the low-dose setting and 629 mGy.cm^2^ for the high-dose setting (compared to a conventional dental panoramic that delivers about 110 mGy.cm^2^). Voxel size was 90 μm x 90 μm x 90 μm.

The 4 hemi-maxillary segments of the maxilla and the 4 hemi-maxillary segments of the mandible from skulls 1 and 2 were all scanned under low and high dose settings. Imaging produced a total of 36 cuts in low-dose and 36 cuts in high-dose imaging; 12 maxillary, 12 of the anterior mandible and 12 of the posterior mandible. Imaging took into account the necessity to include anatomical landmarks used in the treatment planning, especially the location of the inferior alveolar nerve and the maxillary sinus.

### 2.3 Data Collection

A panel of 5 dentists with at least 5 years of experience in oral surgery and implantology were selected for the study. The observers were presented with all 36 images in random order using the software Carestream Health (Carestream 3D Imaging). Following observation of each cut, the observers were asked to answer two different types of questions: 1) quantitative questions that required taking measurements on the observed images and 2) qualitative questions with limited answer choices regarding their perceptions of the observed images.

**Fig. (1):**
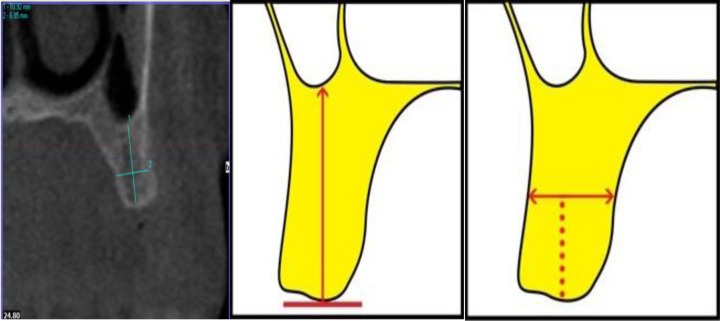
Para-axial view of a maxillary CBCT and schematized diagram of the maxillary region with measurements in the vertical (M1) and horizontal (M2) planes.

The questionnaires provided to the observers included detailed descriptions of the measurements to be undertaken along with schematic illustrations (**Appendix** **1** and **2**). On the maxillary images, the observers were asked to measure alveolar bone height (M1) by drawing a vertical line from the most protruding point on the alveolar crest and alveolar bone width (M2) by drawing a perpendicular bisecting the vertical line (Fig. **1**).

**Fig. (2):**
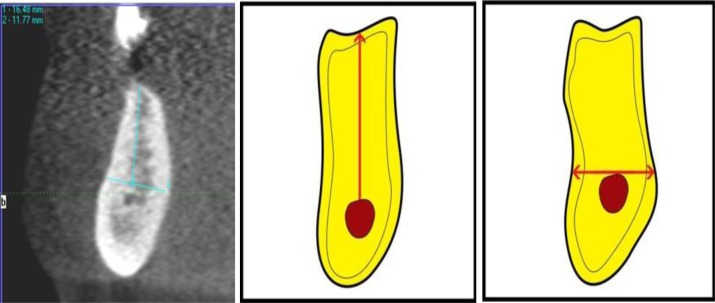
Para-axial view of a mandibular posterior CBCT and schematized of the mandibular region with measurements in the vertical (M3) and horizontal (M4) plane.

In the posterior mandibular area, the observers were asked to measure alveolar bone height as a vertical line form the highest point on the inferior alveolar canal opening to the alveolar crest ridge (M3) whereas alveolar width was to be measured using a tangent to the superior border of the mandibular canal perpendicular to the vertical (M4) (Fig. **2**).

The quantitative measurements were followed by a 4-question structured questionnaire for the maxillary and anterior mandibular images and an additional 3 questions for the posterior mandibular region (Table **1**; **Appendix** **1** & **2**). To avoid deterioration in observers’ concentration, evaluation sessions were limited to 20 minutes and the evaluators were informed beforehand.

### 2.4 Statistical Analyses

Statistical analysis was divided into two parts: 1) analysis of qualitative variables (scores) and 2) analysis of quantitative variables (measurements).

Descriptive statistics were generated for the observers’ responses to the quantitative measurements (M1, M2, M3, and M4). Normality testing showed that the data were non-normally distributed, therefore median values and interquartile ranges of the measurements were calculated for low dose and high dose images separately. Median differences between measurements performed on low dose images and those performed on high dose images were calculated, and the Wilcoxon signed Rank test was used to test for the presence of differences in measurement between the two images qualities. Variability between observers was then analyzed by calculating the concordance correlation coefficient (Lin's coefficient) which assesses the accuracy between observers by measuring the variation of the linear relationship adjusted to the right 45 degrees through the origin and accuracy by measuring how far each observation deviates from the fitted line.

Frequency distributions were generated for all qualitative variables (Q1, Q2, Q3, Q4, Q5, Q6, and Q7) based on low dose images and high dose images. Among the quantitative measures, Q4 and Q5 were considered to be the most relevant to treatment planning and decision making and frequency distributions were generated for the low dose and high dose images. For categorical responses with more than two options, the answers were re-categorized into two groups: 1) sufficient (very sufficient/sufficient) and 2) insufficient (insufficient/very insufficient). Percent agreement and Kappa coefficients were used to assess inter-rater reliability between responses based on low dose images and responses by the same observers based on high dose images and chi-square tests of association were used to assess the presence of statistical differences between the two responses.

All statistical analyses were performed using STATA software 13.0 (Stata Corporation 4905, Lakeway Drive College Station, TX 77845 USA) at the Clinical Investigation and Innovation Unit of the University Hospital Center at Grenoble (France).

## 3 RESULTS

### 3.1 Quantitative Measurements

There were no statistically significant differences between measurements recorded from low dose images and those recorded from high dose images (*p* > 0.05; Table **2**). Concordance correlation coefficients (Lin's coefficients) were all greater than 0.9, suggesting excellent correlation between observers.

### 3.2 Qualitative Measurements

Percent agreement and Kappa measures for questions 4 and 5 were relatively high (Table **3**), especially for raters 1 and 4 when answering question 4. For example, for the first observer (S1), we find that there is a complete agreement between the high dose and low doses in 92% of responses to the question Q4. In addition, the Chi ^2^ test showed that, for almost all observers, there is independence between the answer and the type of image (low or high dose) (Table **4**).

As for Kappa values, they show that the 5 observers (5 seniors) do not respond the same way to questions Q4 and Q5 each dose. For example, the kappa (K = 3.4%) at low doses for the question Q4 and becomes low 71.8% in high doses for the Q5. This means that increasing the dose leads to a higher consistency (Table **5**).

## 4 DISCUSSION

Several studies have illustrated that pre-surgical assessment of bone quality and quantity is essential for successful treatment with dental implants [7, 8]. However, high image quality is not essential for all diagnostic tasks, and the ICRP emphasizes that the visibility of images achieved at higher radiation doses is not necessarily better than that achieved at lower doses [9]. Effective doses for CBCT devices exhibit a wide range with the lowest dose being almost 100 times less than the highest dose. Significant dose reduction can be achieved by adjusting operating parameters including exposure factors and by reducing the Field of View (FOV) to the actual region of interest [10]. Although lower exposure parameters may be associated with lower image quality and diagnostic value, this may not be as significant in the oral and maxillofacial region because the relatively high contrast between the structures of interest makes them less susceptible to changes in the images obtained. Protocols for dose reduction, however, are limited [11].

Our aim was to evaluate the effect of dose reduction on image quality and its diagnostic value for dental implant placement purposes. Studies have been conducted on the influence of varying exposure parameters on image quality, but without analyzing the effect of the adjustment of milliamperage as an isolated factor [12]. The relatively high degrees of concordance observed between low and high dose images in our study suggests an almost perfect match between the two imaging modalities, questioning the need for higher patient radiation exposure. Several studies have described that in more than 50% of reports in the literature, there was a significant reduction in effective dose without actually evaluating the effect of this reduction. Some authors have reported associations between dose reduction and increased image noise, which may degrade and reduce image quality [13, 14].

Using both objective and subjective measures, our results suggest that the expert observers were able to retrieve the information needed from the CBCT cuts independent of the image quality (high or low dose). Our data supports the conclusions of multiple previous researchers regarding the possibility for significant patient dose reduction in CBCT examinations for implant site evaluation without loss of diagnostic information [12, 15, 16]. By evaluating the image quality parameters for the CBCT equipment, Kwong *et al*. (2008) assert that the presence or absence of a filter and the kilovolt (peak) did not affect overall image quality. Images taken at lower milliamperage settings showed good diagnostic quality [16]. Similarly, Vasconcelos *et al*. (2014) concluded that significant dose reduction can be achieved with sufficient diagnostic quality for the planning of implant placement using CBCT images taken with reduced milliamperage settings [17]. In their study of dry mandibles, the authors produced CBCT images under seven different mA settings between 2 and 15 mA and concluded that increasing dosage beyond 6.3 mA did not produce any significant or reduction in image degradation improvement in image quality [17]. While their results support our findings that quantitative maxillary and mandibular measurements recorded from high and low dose images are comparable, their data may also explain our results with respect to the mandibular posterior region. In terms of specifically the mandible, the higher dose setting used in our study (6.3 mA) is similar to the optimal dose recommended by Vasconcelos *et al*. in the mandible and this is perhaps why with respect to the kappa coefficients for qualitative measurements regarding the posterior mandible, our data suggests that the higher the dose, the higher the concordance in subjective image evaluation. Differences in CBCT machines, kilovoltage settings used and voxel size of course limit comparability and suggest that, in fact, the images produced under 6.3 mA in our study are probably different from those produced under 6.3 mA by Vasconcelos and co-workers, but both results suggest that the lowest mA settings may not be sufficient for planning implant placement specifically in the posterior mandibular region. This conclusion is further supported by previous work suggesting that the structures in the posterior region, especially in the mandible, are subjected to greater image quality degradation with low dose protocols compared to anterior structures and this is likely the result of increased bone density in mandibular posterior regions [11]. Nonetheless, while our results suggest that the lowest dosage settings may not always be sufficient for qualitative assessment they do appear to be sufficient for bone measurements, and, if dosage is to be increased then a moderate increase to 6.3 mA (as opposed to high dose settings of 10-15 mA) produces very good reliability of qualitative measurements.

The diagnostic use of oral radiology is an essential part of daily dental practice. The advancement of three-dimensional imaging techniques has resulted in a large group of dentists being confronted with the transition from conventional to digital images, with all its challenges and changes in daily practice [18]. In light of the potentially harmful nature of ionizing radiation and the large variation in patient doses and image quality delivered by different CBCT models and combinations of settings, a patient radiation dose monitoring system is crucial for dental clinics in order to ensure patient safety. However, the variability in image quality and radiation dosage between manufacturers both limits comparability between studies assessing radiation reduction protocols and questions the ability to extrapolate results from research using specific CBCT machines to general clinical practice. Future research in collaboration with clinicians, the CBCT manufacturing industry and radiologists is needed to ensure format convertibility for an efficient and comparable dose monitoring system [19]. While there is still a long way to go before we understand the true value of CBCT in dentistry [20, 21], and in different surgical applications [22-25], the authors recommend that until more universal information is available, clinicians and radiologists must take it upon themselves to ensure that their patients are not being exposed to CBCT radiation beyond was is diagnostically needed for dental treatment planning.

## CONCLUSION

Patient radiation exposure may be significantly reduced without affecting diagnostic image quality when using CBCT imaging for the pre-surgical treatment planning for dental implant placement by reducing milliamperage. Planning implant placement in the posterior mandible may require higher image dosage, but still in the lower range of mA settings provided by CBCT machines. Imaging protocols must be adjusted according to each CBCT machine’s characteristics until future research provides comparable data and universal guidelines become available.

## APPENDIXES

### Appendix 1

- From the most definite point of the ridge, draw the vertical to measure the height of the ridge: ........... mm (M1)

- From the middle of the previous measurement, draw the perpendicular to measure the width of the ridge: ......mm (M2)

- Q1-The top of the alveolar crest is:
- Very good visible / Good visible / Few visible / Very few visible
- Q2-Buccal and palatal cortical are:
- Very good visible / Good visible / Few visible / Very few visible
- Q3-Estimated bone density at the implant site: D1 / D2 / D3 / D4
- Q4-To schedule an implant, the quality of the image appears:
- Very sufficient / Sufficient / Insufficient / Very insufficient

## Appendix 2

- From the most prominent point of the dental canal, draw the vertical to measure the bone height above the dental canal: ……… mm (M3)
- Q5-The mandibular canal is:
- Very good visible / Good visible / Few visible / Very few visible
- Q6-Do you need to use the sagittal or panoramic slices to identify the mandibular canal? Yes / No
- Q7-Have you tried to move forward and backward in the panoramic sections to locate the mandibular canal? Yes / No
- Draw a horizontal line tangent to the upper edge of the dental canal to measure the width of the ridge: … mm(M4)
- Q1-The top of the alveolar crest is:
- Very good visible / Good visible / Few visible / Very few visible
- Q2-Buccal and palatal cortical are:
- Very good visible / Good visible / Few visible / Very few visible
- Q3-Estimated bone density at the implant site: D1 / D2 / D3 / D4
- Q4-To schedule an implant, the quality of the image appears:
- Very sufficient / Sufficient / Insufficient / Very insufficient

## Figures and Tables

**Figure:**
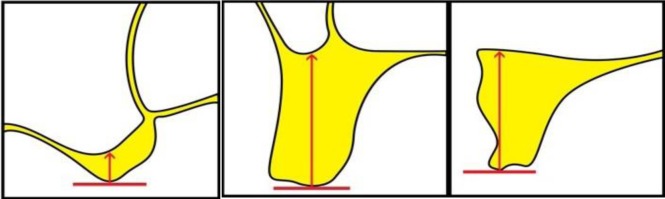


**Figure:**
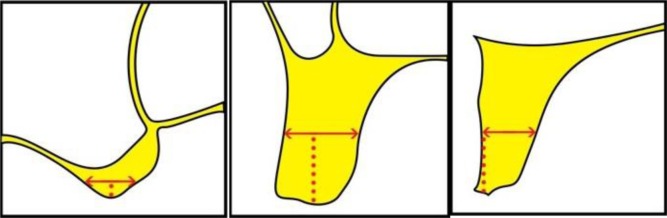


**Figure:**
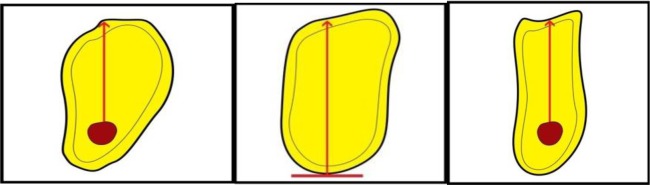


**Figure:**
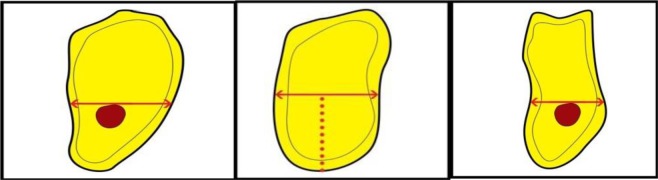


**Table 1:** Questions answered by observer panel with respect to CBCT image cuts.

No.	Question	Possible Answers
Q1	The visibility of the top of the alveolar crest is:	Very good / good / poor / very poor
Q2	The visibility of the buccal and palatal cortical plates is:	Very good / good / poor / very poor
Q3	The estimated bone density at the implant site is:	D1/ D2/ D3/ D4
Q4	For the purpose of implant placement, the quality of the image appears to you as:	Very sufficient/ sufficient/ insufficient/ very insufficient
Q5*	The visibility of the mandibular canal is	Very good / good / poor / very poor
Q6*	Do you need to use the sagittal or panoramic slices/cuts to identify the mandibular canal?	Yes/ No
Q7*	Have you tried to move forward and back in panoramic slices/cuts to locate the mandibular canal?	Yes/ No

* Questions 5, 6 and 7 were answered only for images of the posterior mandible

**Table 2:** The medians of the differentials between low dose and high dose for quantitative variables.

Median of Differential LD-HD	M1	M2	M3	M4
S1	-0.05 [-15; 11.8]	-0.25 [-1.7; 0.9]	-0.3 [-2.5; 0.6]	0 [-0.5; 1.6]
	0.989	0.057	0.107	0.475
S2	-0.05 [-5.5; 0.9]	-0.1 [-3; 1]	0.05 [-0.6; 0.7]	0 [-0.3; 0.4]
	0.106	0.096	0.974	0.551
S3	-0.1 [-5.3; 2.3]	0.1 [-1.7; 1]	-0.2 [-1.1; 0]	-0.1 [-0.6; 0.9]
	0.920	0.087	0.409	0.906
S4	-0.1 [-4.9; 1.2]	0 [-1.1; 1.5]	-0.05 [-1.1; 0.3]	0.1 [-3.5; 0.8]
	0.085	0.396	0.305	0.528
S5	0 [-5.1; 1.3]	-0.5 [-1.1; 1.3]	-0.15 [-0.9; 0.9]	-0.2 [-2 ; 0.4]
	0.510	0.456	0.430	0.077

**Table 3:** Quantification of degree of concordance (intra-observer variability).

Concordance and Test between HD and LD	Q4 N=36	Q5 N=12
S1	91.67%	75%
	1.000	0.222
S2	61.11%	41.67%
	0.159	1.000
S3	77.8%	58.33%
	0.488	0.470
S4	91.67%	50%
	1.000	1.000
S5	58.33%	66.7%
	0.47	0.545

**Table 4:** Concordance and *chi^2^* test for qualitative variables.

Concordance and Test	Q1 N=36	Q2 N=36	Q3 N=36	Q6 N=12	Q7 N=12
S1	83.3%	94.4%	66.7%	83.3%	50.0%
	0.370	0.110	0.049	1.000	0.509
S2	83.3%	83.3%	94.4%	58.3%	58.3%
	0.310	0.044	<0.001	0.470	0.470
S3	83.3%	69.4%	86.1%	58.3%	58.3%
	1.000	0.064	<0.001	0.576	0.576
S4	66.7%	77.8%	75.0%	58.3	50.0%
	1.000	0.207	0.020	0.470	1.000
S5	72.2%	80.6%	72.2%	83.3%	83.3%
	0.014	0.001	0.017	0.061	0.061

**Table 5:** Kappa coefficient inter- observers for Q4 and Q5 issues

**Kappa**	**Q4** **N=36**	**Q5** **N=12**
Lower dose	7.1%	28.7%
High dose	33.4%	71.8%
